# The Relationship Between Alexithymia and Type 2 Diabetes: A Systematic Review

**DOI:** 10.3389/fpsyg.2020.02026

**Published:** 2020-08-28

**Authors:** Gabriella Martino, Andrea Caputo, Carmelo M. Vicario, Antonino Catalano, Peter Schwarz, Maria C. Quattropani

**Affiliations:** ^1^Department of Clinical and Experimental Medicine, University of Messina, Messina, Italy; ^2^Department of Dynamic and Clinical Psychology, Sapienza University of Rome, Rome, Italy; ^3^Department of Cognitive Sciences, Psychology, Education and Cultural Studies, University of Messina, Messina, Italy; ^4^Department of Medical Endocrinology, Copenhagen University Hospital, Rigshospitalet, Copenhagen, Denmark

**Keywords:** alexithymia, psychological distress, type 2 diabetes mellitus, metabolic syndrome, chronic disease

## Abstract

**Background:** This systematic review analyzed the relationship between alexithymia, considered as the inability to recognize and express thoughts and emotions, and type 2 diabetes mellitus (T2DM), one of the most common chronic illness, characterized by a metabolic disorder burdened by high morbidity and mortality worldwide due to its outcomes.

**Methods:** PRISMA guidelines were followed throughout this systematic review of the recent literature indexed in the databases PubMed, PsycInfo, Scopus, and Web of Science. Search terms for eligible studies were: “Type 2 diabetes” OR “T2DM” AND “Toronto Alexithymia Scale” OR “TAS-20”[All Fields].

**Results:** The initial search identified 61 indexed scientific publications. After screening we found that seven publications met the established scientific inclusion and exclusion criteria. It emerged that alexithymic patients ranged from 25 to 50% across the examined publications and it appeared that patients with T2DM generally reflected greater values of alexithymia, revealing particular differences among TAS domains. Moreover, emlpoyed participants were alexithymic to a greater extent compared to non-working participants (77.8 vs. 35.4%) and alexithymia was 2.63 times more severe among working participants when examining predictors of alexithymia. When evaluating the correlations between alexithymia and HbA1c or fasting blood glucose levels we found strong associations equal to 0.75 and 0.77 for TAS-20 total scores, respectively. While alexithymic participants showed significantly higher levels of HbA1c and blood glucose when compared to the non-alexithymic participants.

**Conclusions:** The results of this systematic review of the current literature highlight the need of alexithymia evaluation in patients with T2DM. The high prevalence in T2DM and strong associations with poorly regulated diabetes and psychological distress, indicate a significant relationship between poor glycemic control and psychological distress, such as anxiety and depression, and quality of life. Further studies are needed focusing on age and gender differences in order to be able to improve clinical psychological care and prevention.

## Introduction

Alexithymia is considered as the inability to distinguish between emotions, thoughts, and physiological responses to stimuli, involving difficulties in recognizing and verbalizing emotions and externally oriented thinking (Nemiah and Sifneos, 1970; Sifneos, 1996; Taylor and Bagby, 2000; Tordeurs and Janne, 2000). Alexithymia is also defined as a personality trait which manifests itself in comorbidity with different physical and psychopathological disorders (Lumley et al., 2005, 2007; Mattila et al., 2009; Honkalampi et al., 2010; Tolmunen et al., 2011; Marchetti et al., 2019) and retained as a transient variable state related to both psychopathological conditions and stress levels (Pollatos et al., 2011). Also, alexithymia seems to be involved in the pathogenetic mechanism of several somatic illness (Porcelli et al., 1996; Willemsen et al., 2008; Mazaheri et al., 2012; Talamonti et al., 2016; Marchi et al., 2019; Martino et al., 2020c) and it appears to be associated with depression and anxiety in patients with type 2 diabetes (T2DM) (Friedman et al., 2003; Luminet et al., 2006; Pouwer et al., 2010; Sapozhnikova et al., 2012; Shinkov et al., 2018). Alexithymia and psychological distress, as anxiety and depression, may impair patient's compliance and adherence, concurring in a worst clinical presentation and course of a chronic disease (Sapozhnikova et al., 2012; Hintistan et al., 2013; Mnif et al., 2014; Stanton and Hoyt, 2017; Marchini et al., 2018; Settineri et al., 2019; Vicario and Martino, 2020; Vicario et al., 2020; Vita et al., 2020).

T2DM is a common chronic illness and it is considered a major health concern due to its prevalence and outcomes. T2DM may involve a serious impact on both morbidity and mortality, compromising individuals' health and perceived quality of life and provoking a very thoughtful social burden to society (Perry et al., 2015; Craparo et al., 2016; Catalano et al., 2019; Conversano, 2019; Di Giuseppe et al., 2019, 2020; Martino et al., 2019c, 2020b; Merlo, 2019; Zimmerman et al., 2019; Lenzo et al., 2020). T2DM is a pandemic chronic metabolic disease, due to insulin deficit and resistance, which affects more than 280 million people worldwide and within the next decade it is expected that more than 430 million people will suffer T2DM (American Diabetes Association, 2018).

T2DM is a chronic disease with severe complications if poorly controlled and could in turn compromise psychological balance (Whithorth et al., 2016). It is hypothesized that low glycemic control affects brain and mental functions leading to psychological distress, which is shown to be more prevalent in T2DM patients than in the general population (Lustman and Clouse, 2005; Bahar et al., 2006; Collins et al., 2009; Lee et al., 2009; Papelbaum et al., 2010; Sulaiman et al., 2010), as even results by the increased risk of mental disorders (Anderson et al., 2001; Collins et al., 2009; Lemche et al., 2014). Particularly, some studies revealed that compliance and adherence are crucial factors to adequately manage such chronic disease (Marchini et al., 2018; Martino et al., 2019b).

It is well-known that poor metabolic control in T2DM leads to several severe and life threatening complications as among other cardiovascular diseases (Li et al., 2008; American Diabetes Association, 2018; Guicciardi et al., 2019). However, poor metabolic control is also associated with increased risk of psychopathological conditions, including also alexithymia (Abramson et al., 1991; Luminet et al., 2006).

Focusing the opposite way, patients suffering from alexithymia and T2DM have a significant metabolic imbalance and control, which might be explained by difficulty in recognizing their body signals, perceptions, and emotions (Abramson et al., 1991; Sapozhnikova et al., 2012; Hintistan et al., 2013; Lemche et al., 2014; Luca et al., 2015; Rosa et al., 2019; Orrù et al., 2020). Moreover, the failure to recognize body symptoms and emotion perceptions could lead to a further incomprehensible psychological and physical suffering, due to poorly regulated T2DM, which may impair the ability to manage their diabetes (Friedman et al., 2003).

Thus, it is crucial to both elaborate and plan a deep clinical psychological investigation together with adequate medical evaluation in order to find psychological features, such as alexithymic patients, in favor of the best health course and specific treatments. Therefore, the aim of this systematic review is providing the current insights on the alexithymic characteristics of patients with T2DM and highlighting the clinical faces of this complex. Our goal is to improve knowledge on the complex of alexithymia, T2DM and other related factors, which may support both psychologist and physicians to realize strategies and interventions to promote the adequate managing of T2DM. Better understanding of this complex among patients and health care professionals, might improve patients' knowledge of such chronic illness, way of feeling themselves and perceived quality of life.

## Materials and Methods

### Data Source and Search Strategy

The review was performed according to the Preferred Reporting Items for Systematic Reviews and Meta-Analyses (PRISMA) (Liberati et al., 2009; Moher et al., 2009). PubMed, PsycInfo, Scopus, and Web of Science databases were searched in March 2020 for eligible studies between 2015 and 2020, and the following combination of search terms was employed: “Type 2 diabetes^*^” OR “T2DM” AND “TAS-20” OR “Toronto Alexithymia Scale” [All Fields]. The term TAS-20 (or Toronto Alexithymia Scale) was selected to search records providing empirical data about alexithymia, by relying on the most used assessment instrument.

### Publication Screening and Eligibility Criteria

After conducting the first screening we eliminated study duplicates. During the second selection all titles and abstracts were screened and possible relevant studies were identified for full text review by two independent researchers in clinical psychology for eligibility. Inclusion criteria were: Scientific publications in English and with peer review published from 2015 and up to March 2020, adopting quantitative research designs, and reporting relevant and empirical data on alexithymia and T2DM. The limited time range (from 2015 to 2020) was selected because the authors' choice was to review updated literature providing results from very recent publications.

### Analysis Reviewed Publications and Data Synthesis

Methods were performed accordingly to the PRISMA guidelines (Liberati et al., 2009; Moher et al., 2009), considering that the heterogeneity of the explored research designs was not appropriate to be explored by a meta-analysis. To avoid the risk of bias, researchers in clinical psychology reviewed independently the articles to determine the adequate reliability of the used methodology. Data were extracted into an Excel spreadsheet, with bibliographic information and study specific details were recorded, such as sample characteristics (e.g., age, gender, sample size, presence of comparison groups) and scores of TAS-20 and further used measures. Selected studies were considered by comparing significant data and identifying the indexes of the measured variables, to provide a qualitative synthesis. Discrepancies on inclusion/exclusion criteria and study characteristics were solved by consensus.

## Results

### Search Result

**Figure 1:**
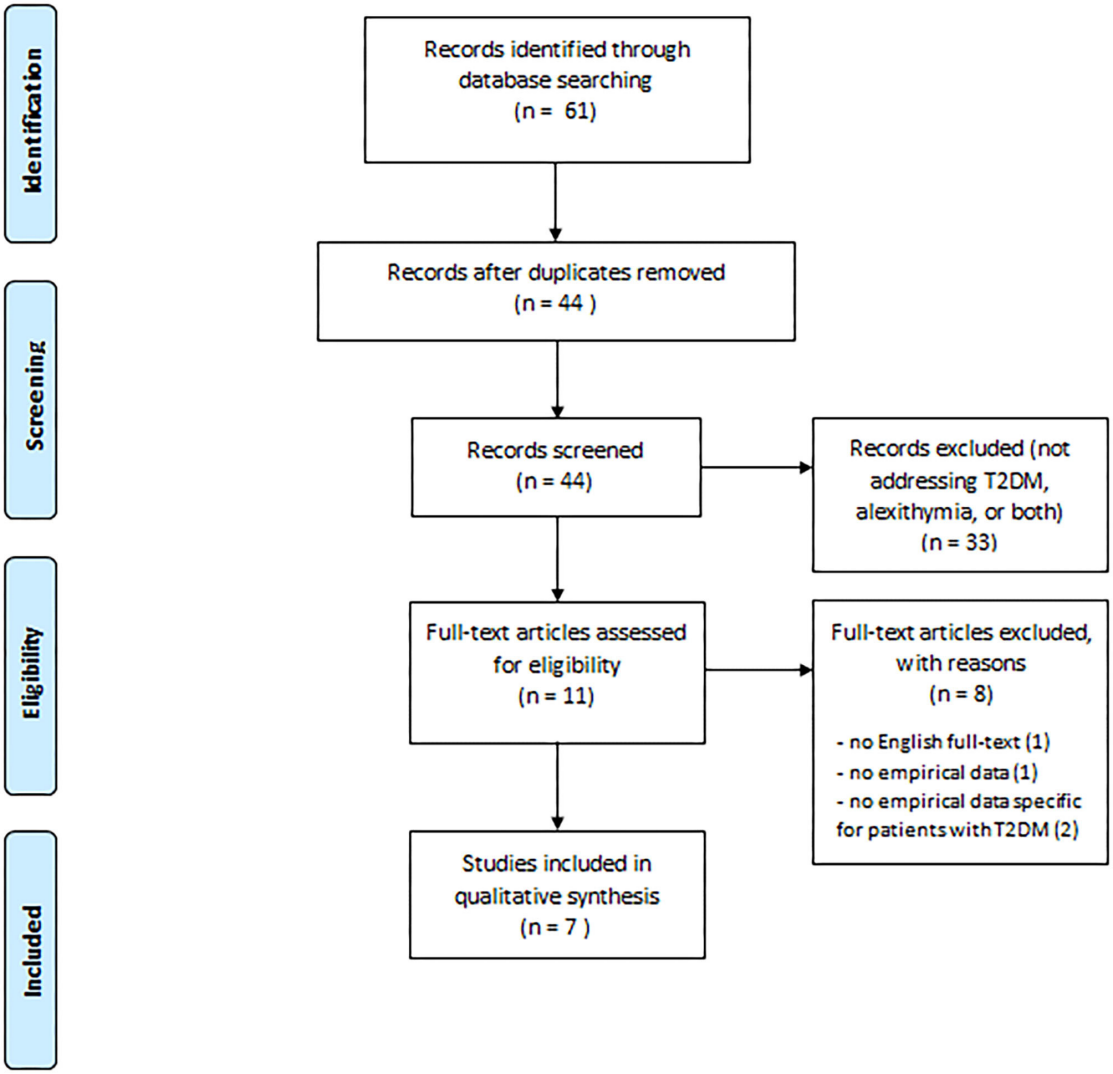
PRISMA flow chart of the systematic search.

Figure 1 shows our search result and screening results according to PRISMA. Our search identified 61 publications. Seventeen publications were duplicates leaving our search with 44 publications for title and abstract review. After this review process we identified in total 11 papers for full review. The excluded publications did not fulfill the inclusion criteria as 23 did not address T2DM, one did not address alexithymia and nine did not address any of the two. Thus, the remaining 11 publications were full text reviewed and four of them were removed for the following reasons: one did not provide English full-text (Shayeghian et al., 2015), one did not provide empirical data (Melin et al., 2016), and two were more widely addressed to patients with diabetes without providing specific or disaggregated results about patients with T2DM (De Oliveira Regina and Tambascia, 2017 Marchini et al., 2018). We concluded that seven papers could be included in our systemic review based on the inclusion criteria.

### Study Characteristics

We found that most included studies were conducted in Italy (three out of seven), and pertained to cross-sectional research studies. Specifically, four studies adopted a one-sample research design (Luca et al., 2015; Avci and Kelleci, 2016; Lai et al., 2019; Martino et al., 2019a), whereas three studies also included comparison groups referring to healthy controls (Shahi and Mohammadyfar, 2017; Fares et al., 2019) or patients with T1DM (Melin et al., 2017). Five studies reported specific inclusion/exclusion criteria for patient selection (Avci and Kelleci, 2016; Melin et al., 2017; Fares et al., 2019; Lai et al., 2019; Martino et al., 2019a). The most reported inclusion criterion was having good language proficiency to fully comprehend or perform the psychological administration (Melin et al., 2017; Fares et al., 2019; Lai et al., 2019; Martino et al., 2019a). Three studies also reported time since diagnosis as a further criterion, including patients with a diagnosis since at least 6 months (Avci and Kelleci, 2016), 1 year (Lai et al., 2019), or 5 years (Martino et al., 2019a). Whereas, the most frequent exclusion criterion was lack of cognitive or psychiatric disorders (Avci and Kelleci, 2016; Melin et al., 2017; Fares et al., 2019; Lai et al., 2019; Martino et al., 2019a). Concerning sample size, the retrieved studies included a number of patients with T2DM ranging from 24 (Melin et al., 2017) to 326 (Avci and Kelleci, 2016), with an average of 108 patients (*SD* = 102). Overall, the patients were aged 27–90 years (mean age 63 years). Gender was fairly well-distributed in the considered studies, with a mean of 53% of male patients (average of 36.2–68.7% in the studies). Three studies (Luca et al., 2015; Fares et al., 2019; Lai et al., 2019) reported patient diabetes duration which in general was above 10 years. Besides, patients' diabetes complications were reported in three studies (Avci and Kelleci, 2016; Fares et al., 2019; Martino et al., 2019a) and diabetes-related treatment information was provided in more than half (Luca et al., 2015; Avci and Kelleci, 2016; Melin et al., 2017; Fares et al., 2019). The main characteristics of the studies are reported in Table 1.

**Table 1:** Included items characteristics.

**Authors**	**Year**	**Journal**	**Country**	**Purpose**	**Number of patients with T2DM (% males)**	**Age range or mean age (SD)**	**Presence of comparison groups and sample size**
Fares et al.	2019	Journal of Diabetes and Metabolic Disorders	Lebanon	Evaluate the prevalence of alexithymia and its relationship with glycemic control among Lebanese adults with T2DM compared to healthy controls	104 (60.6%)	29–90 years; 59.4 (14.2)	Yes (100 healthy controls)
Lai et al.	2019	European Review for Medical and Pharmacological Sciences	Italy	Assess the association among alexithymia, anxiety, depression, stress, and social support with adherence and healthcare spending in type 2 diabetic patients	64 (68.7%)	55–90 years; 67 (11)	No
Martino et al.	2019	Mediterranean Journal of Clinical Psychology	Italy	Investigate the relationship between alexithymia and affective dimension such as anxiety and depression levels, and health related quality of life in patients with T2DM	47 (36.2%)	55–75 years; 65.3 (5.9)	No
Melin et al.	2017	BMC Psychiatry	Sweden	Compare the clinical presentation of patients with T1DM and T2DM in relation to self-reported depression, anxiety, alexithymia, obesity, and midnight salivary cortisol (MSC)	24 (50%)	31–59 years	Yes (148 patients with T1DM)
Avci and Kelleci	2016	Patient Preference and Adherence	Turkey	Determine the prevalence of alexithymia in patients with T2DM and the factors affecting it, such as anxiety and depression	326 (47.5%)	62.0% were in the ≥65 years age group	No
Shahi and Mohammadyfar	2016	Personality and Individual Differences	Iran	Compare depression, anxiety, stress, quality of life, and alexithymia in people with T2DM and their non-diabetic counterparts	60 (50%)	57.8 (10.5)	Yes (60 healthy controls)
Luca et al.	2015	Journal of Endocrinological Investigation	Italy	Assess the prevalence of depression and alexithymia among patients with T2DM and investigate the possible correlations between these psychopathological phenomena and glycemic control	128 (58.6%)	64.8 (11.2)	No

### The Prevalence of Alexithymia in Patients With Type 2 Diabetes Mellitus

With the exception of two of the seven studies (Shahi and Mohammadyfar, 2017; Lai et al., 2019), information about the TAS-20 total score was provided. The mean value of alexithymia ranged from 52.2 (Fares et al., 2019) to 60.5 (Martino et al., 2019a), thus falling in the range of intermediate/borderline alexithymia, which is comprised between 52 and 60 points. In four studies, the percentage of alexithymic patients (≥61) was also indicated (Luca et al., 2015; Avci and Kelleci, 2016; Melin et al., 2017; Fares et al., 2019), ranging from 25% (Melin et al., 2017) to 50% out of the total sample (Luca et al., 2015). Finally, three studies compared alexithymia of patients with T2DM with healthy controls (Shahi and Mohammadyfar, 2017; Fares et al., 2019) or patients with T1DM (Melin et al., 2017) and showed statistically significant differences, with patients with T2DM generally having greater TAS-20 values of alexithymia or likelihood to be alexithymic based on the accepted cutoff.

Concerning the three subscales of TAS-20 (Difficulty Identifying Feelings, Difficulty Describing Feelings, Externally-Oriented Thinking), we calculated effect sizes (ES) (expressed as the mean divided by the standard deviation of the sample) for each study providing descriptive statistics of the alexithymia dimensions (Luca et al., 2015; Avci and Kelleci, 2016; Shahi and Mohammadyfar, 2017; Fares et al., 2019; Martino et al., 2019a). This was to compare the mean values on the different subscales. On average, patients scored higher on Externally-Oriented Thinking (ES = 5.04) compared to both Difficulty Describing Feelings (ES = 4.10) and Difficulty Identifying Feelings (ES = 3.20), with the latter having the lowest mean values.

### Alexithymia and Socio-Demographic Characteristics

Two studies specifically evaluated gender differences in patients with T2DM. Particularly, Avci and Kelleci (2016) found alexithymia was more severe in females compared to male participants (45.6% of females were alexithymic vs. 29% of males). As well, multivariate analysis of factors affecting alexithymia did not confirm gender as a statistically significant predictor. Conversely, Fares et al. (2019) showed male participants were more likely to present alexithymia than females (46.0 vs. 19.5%, respectively), although multivariate analysis of alexithymia predictive factors revealed that gender did not show significant.

With regard to age differences, two publications showed no statistically significant differences based on either the patients alexithymic status (Luca et al., 2015; Avci and Kelleci, 2016) or the inspection of potential predictors of alexithymia (Avci and Kelleci, 2016).

The study by Avci and Kelleci (2016) examined further socio-demographic information in relation to alexithymia, such as participants' education level, marital status, employment status, and perceived income level. In general, the publications showed that working participants were alexithymic to a greater extent than unemployed ones (77.8 vs. 35.4%) and alexithymia was 2.63 times more severe among employed participants when examining alexithymia predictors, whereas no relations were found on other considered socio-demographic variables.

### Alexithymia and Lifestyle

Two studies inspected the relationship between alexithymia and lifestyle factors. Specifically, the study by Fares et al. (2019) found that patients with alexithymia had higher frequencies of alcohol consumption (75.7 vs. 37.3%), lower rates of physical activity (27 vs. 50.7%), and fewer self-reports of adequate dietary intake related to diabetes (40.5 vs. 71.6%). Whereas, no significant association with tobacco and body mass index (BMI) was observed. However, such factors did not constitute significant predictors of alexithymia in multivariate analysis. Furthermore, the study by Lai et al. (2019) observed that TAS-20 difficulties in identifying and describing feelings were negatively associated with physical activity with low effect sizes (*r* = −0.26 and *r* = −0.25, respectively).

### Alexithymia, Diabetes Duration, and Treatment Information

Two studies examined the relationship between alexithymia and diabetes duration. Avci and Kelleci (2016) found a greater rate of alexithymia among patients who had diabetes ≥11 years, but diabetes duration was not a reliable predictor of alexithymia. On the other hand, the study by Luca et al. (2015) showed that alexithymic patients had, on average, longer diabetes duration (13.5 vs. 10.3 years) despite not to a statistically significant extent.

The association of alexithymia and treatment-related variables was also considered. Avci and Kelleci (2016) did not find differences between alexithymic and non-alexithymic patients based on type of treatment (insulin + diet vs. oral antidiabetic + diet), which did not result in a significant predictive factor of alexithymia. Consistently, the study by Luca et al. (2015) did not show differences in diabetes treatment (diet, per oral anti-diabetics, or subcutaneous insulin) based on patients' alexithymic status.

### Alexithymia, Glycemic Control, and Hospitalization

Three studies examined the potential association between alexithymia and glycemic control. The study by Fares et al. (2019) assessed the impact of alexithymia on glycemic control based on values of HbA1c and fasting blood glucose levels. Correlations of alexithymia with HbA1c and fasting blood glucose levels showed a strong association equal to 0.75 and 0.77 for TAS-20 total scores, respectively (and ranging from 0.63 to 0.75 and from 0.67 to 0.77 at the relative subscales). Besides, alexithymic patients showed significantly higher levels of HbA1c and blood glucose levels as compared to not-alexithymic patients; specifically, those having HbA1c >7% were more likely to have alexithymia in comparison to their counterparts. The study by Avci and Kelleci (2016) confirmed a high correlation between alexithymia and HbA1c (equal to 0.81) and a greater rate of alexithymic patients among those having HbA1c levels ≥7% (44 vs. 26.9% of those with normal values of glycemic control). Then, alexithymia was found 2.09 times higher among patients with HbA1c ≥7.0% compared to their counterparts. The study by Luca et al. (2015) detected a statistically significant but modest correlation (*r* = 0.30) between HbA1c and TAS-20 factor about identifying feelings. HbA1c was observed higher in alexithymic patients compared to non-alexithymic ones. Further, patients with pathological high HbA1c had higher TAS-20 and subscale scores compared to patients with HbA1c within normal range.

Besides this, hospitalization due to acute and chronic diabetes complications was considered by two studies. Lai et al. (2019) showed that TAS-20 total and externally oriented thinking scores were positively correlated with the number of hospitalization days and that alexithymia was predictive of a substantial increase in hospitalization. In line with this, the study by Fares et al. (2019) found that patients with alexithymia had hospitalizations for hyperglycemia (during the past year) ~5 times more frequent than their counterparts (82.9 vs. 17.1%) and that hospitalization represented a significant predictor of alexithymic status.

### Alexithymia and Psychological Distress

#### Alexithymia and Depression

The association with alexithymia and further variables regarding the psychological status were evaluated. Four studies considered depression levels (Luca et al., 2015; Avci and Kelleci, 2016; Melin et al., 2017; Martino et al., 2019a). Avci and Kelleci (2016) used the depression subscale of the Hospital Anxiety and Depression Scale (HADS), using the cutoff point ≥8 to determine the risk of depression. A very high correlation emerged between alexithymia and depression levels (*r* = 0.80). Comparing alexithymic and non-alexithymic patients, the authors found a higher rate of participants with clinically significant depression (51.7 vs. 26.3% of participants whose depression subscale score was <8). In more detail, according to the multivariate analysis, alexithymia was 2.57 times more severe in the depressed participants than in their counterparts. The study by Luca et al. (2015) used the Hamilton rating scale for depression (HAM-D) investigating the presence of depressive symptoms, with a score >7 indicating the presence of a depressive condition. Overall, alexithymic patients presented significantly higher depression levels than non-alexithymic patients, both regarding the total score and most of HAM-D subscale scores (i.e., anxiety/somatization, cognitive disturbance, diurnal variation, psychomotor retardation, and sleep). Also, TAS-20 total and subscale scores were higher in depressed patients than in non-depressed patients. Melin et al. (2017) assessed self-reported depression through the relative subscale of the HADS adopting the recommended cut off level ≥8 points. They highlighted that depressed patients had higher prevalence of alexithymia compared to non-depressed patients (67 vs. 11%) and that being alexithymic was a predictive factor for depression risk. Then, Martino et al. (2019a) administered the Beck Depression Inventory-second edition (BDI-II) to evaluate depression levels, which correlated with the TAS-20 total score, to a moderate extent (*r* = 0.33).

#### Alexithymia and Anxiety

Two studies out of the seven assessed publications found association between alexithymia and anxiety. The study by Avci and Kelleci (2016) used the anxiety subscale of the Hospital Anxiety and Depression Scale (HADS), adopting the cutoff point of 11 to determine clinically significant anxiety levels. A high association was detected between alexithymia and anxiety (*r* = 0.65) and a greater rate of patients with anxiety problems was found in alexithymic rather than non-alexithymic patients (61.3 vs. 38.7%). In addition, the results of multivariate analysis showed that alexithymia was 3.77 times more severe in patients whose anxiety score was ≥11. Whereas, the study by Martino et al. (2019a) used the Hamilton Anxiety Rating Scale (HAM-A) to detect anxiety levels, allowing the measurement of two components respectively regarding psychic and somatic anxiety. Strong statistically significant associations were detected between alexithymia and anxiety (*r* = 0.66), both the psychic (*r* = 0.56) and somatic components (*r* = 0.61). With reference to the alexithymia factors, difficulty identifying feelings was strongly associated with overall anxiety levels (*r* = 0.57), regarding both psychic (*r* = 0.33), and somatic anxiety (*r* = 0.57). Moreover, difficulty describing feelings was associated only with HAM-A total score to a moderate extent (*r* = 0.37), whereas no association was found with externally-oriented thinking.

#### Alexithymia and Quality of Life

Two studies focused association between alexithymia and quality of life. Luca et al. (2015) evaluated the quality of life using the Quality of Life Index (QL), as an overall measure pertaining to activity, daily life, health, support, and mood. The authors found that quality of life was lower in alexithymic patients than in non-alexithymic patients. Whereas, the study by Martino et al. (2019a), evaluated perceived health-related quality of life using the Short Form Health Survey (SF-36) questionnaire, which provided two indexes regarding physical and mental well-being (*PCS*, physical component summary and *MCS*, mental component summary, respectively). Their findings highlighted that alexithymia was negatively associated with the MCS value to a moderate extent (*r* = −0.41), specifically only with difficulty identifying feelings (*r* = −0.50), while no association was found with the PCS value.

## Discussion

Our study aim was to evaluate the current evidence of T2DM in relation to alexithymia. A limited number of scientific publications are focusing emotional capacities among patients with T2DM, despite the relevance of emotion-management in such chronic disease being a well-acknowledged problem in terms of treatment adherence and health status (Cummings et al., 2014; Gonzalez et al., 2015).

With regard to the prevalence of alexithymia, our findings suggest patients with T2DM show values reflecting possible alexithymic traits. Specifically, among patients with T2DM, it is estimated that those being alexithymic (based on the accepted cutoff for TAS-20) may range from 25 to 50%. The significant prevalence of alexithymia in this specific population is further confirmed by studies using comparison groups composed of healthy controls (Shahi and Mohammadyfar, 2017; Fares et al., 2019) or patients with T1DM Melin et al. (2017). In more detail, among the alexithymia factors, it is worth-noting that externally-oriented thinking shows the highest values. Differently from difficulties identifying or describing feelings, this dimension represents a rigid and concrete cognitive style of thinking which does not necessarily include the emotional experience. It reflects the tendency to focus on external events over inner experiences resulting in poor resources of introspection. From a psychosomatic perspective, along with a limited fantasy life, it relates to the operative thinking observed in patients with physical illnesses (Marty and De M'Uzan, 1963). Besides, it is associated with lower level of body attention, less time spent to body scan, less bodily perceived change, and lower reflexion (Fantini-Hauwel, 2014), thus suggesting the potential enactment of avoidant strategies, in psychodynamic terms (Marchetti et al., 2017; Marchini et al., 2018; Fantinelli et al., 2019; Velotti et al., 2019).

About the relationship between alexithymia and socio-demographic characteristics, the evaluated scientific publications show contrasting observations with regard to gender differences (Avci and Kelleci, 2016; Fares et al., 2019) and no statistically significant results due to the participants' age (Luca et al., 2015; Avci and Kelleci, 2016). Instead, employment status seems to play a crucial role in alexithymia, probably as employed patients may have more problems to both manage and integrate their own disease in everyday life. However, this data is derived from only one study (Avci and Kelleci, 2016) and it needs further scientific investigation.

Some interesting, but not exhaustive, findings refer to the association of alexithymia and lifestyle factors, such as reduced physical activity (Fares et al., 2019; Lai et al., 2019). Since alexithymia may involve a reduced capacity to deal with emotional burden, patients may perceive the stressful rigors of diabetes management to a greater extent without complying with the required lifestyle changes (Dipnall et al., 2015).

Focusing T2DM, disease duration appeared in some but not all studies associated with alexithymia, and T2DM treatment (diet, oral anti-diabetics, and subcutaneous insulin) did not show a significant role based on the empirical observations (Luca et al., 2015; Avci and Kelleci, 2016).

Interestingly, more robust evidence emerges with regard to the association between alexithymia and glycemic control, with generally large-sized effects (Luca et al., 2015; Avci and Kelleci, 2016; Fares et al., 2019). Based on the examined research findings, alexithymic traits represent a potential predictor of poor glycemic control; as well, good glycemic control is found to be protective against the presence of alexithymia. Several explanations may be hypothesized. On one hand poor awareness of bodily sensations in alexithymic patients may negatively impact coping strategies in T2DM management relatively to both disease knowledge and capabilities of self-care (Topsever et al., 2006). Besides, since alexithymia involves scarce emotional competence patients with alexithymic traits may be less prone to recognize their illness and adequately follow the medical regimen, overall showing worse glycemic control (Caputo, 2013; Marchini et al., 2018). On the other hand, poor glycemic control in diabetes could in turn negatively affect body functions, particularly cognitive and emotional processing, because of vascular and neuronal mechanisms, thus resulting in greater alexithymia levels (Contreras and Gutiérrez-García, 2017; Fares et al., 2019). The negative relationship between alexithymia and glycemic control may be supported also in the light of the association between alexithymia and increased hospitalization due to acute and chronic diabetes complications, as suggested by some studies (Fares et al., 2019; Lai et al., 2019).

The current review provides solid empirical evidence across the examined studies also regarding the relationship between alexithymia and psychological distress. In detail, alexithymia is found to strongly correlate with depression levels to a clinically significant extent, generally with moderate or large effect sizes. This could depend on the inherent difficulties dealing with painful emotions of grief and loss, thus preventing the individuals from psychological mourning elaboration, whose depression is a signal both in T2DM and other chronic conditions (Coughlin, 2011; Marchini et al., 2018; Caputo, 2019; Vicario et al., 2019; Martino et al., 2020a). We also confirm evidence of a high correlation between alexithymia and anxiety, especially with the difficulty identifying feelings (Martino et al., 2019a). This could suggest the impaired ability to monitor body signals may prevent from making sense of illness, potentially increasing perceived diabetes-related stress (Knowles et al., 2020). These findings highlight alexithymic patients to have poor quality of life (Luca et al., 2015) and poor mental well-being associated with their perceived health-status (Martino et al., 2019a). In particular, difficulty in identifying feelings seems to play a relevant negative role (Martino et al., 2019a), thus supporting the previously discussed hypothesis about mourning illness (Hyphantis et al., 2005, 2013; D'Alberton et al., 2012; Conti et al., 2016; Marchini et al., 2018; Velotti et al., 2019). Based on the present study findings, psychotherapeutic or psychological interventions could be proposed aimed at fostering patients' diabetes adaptation according to a psychodynamic perspective. Such interventions could work on depressive and anxiety feelings over the course of the disease, which may prevent from accepting illness and enacting consistent self-care behaviors, with negative consequences on treatment adherence and diabetes management (D'Alberton et al., 2012; Marchini et al., 2018). Especially, therapeutic writing and expressive disclosure interventions could facilitate the emotional processing of thoughts and feelings about diabetes, enabling the capacity for introspection and thus ensuring biographical continuity and coherence to the self over the course of the chronic condition (Caputo, 2019). In such a way, it could be possible to contrast potential processes of personification and protagonization of illness in narrative, thus contributing to repair a defective identity that is disrupted by the disease (D'Alberton et al., 2012; Marchini et al., 2018).

This systematic review has some limitations that should be noted. The low number of retrieved articles, even due to the selection of available literature from 2015 to date, which on the other side allowed to highlight results from very recent publications, even in line with the most recent progress in the clinical management of T2DM. There is a strong heterogeneity of the examined studies regarding the adoption of inclusion/exclusion criteria, the size and characteristics of the samples, the diverse data analyses conducted, and the different assessment tools used to inspect other lifestyle and psychological variables. As well, it should be acknowledged that the cross-sectional (rather than longitudinal) design of the examined studies does not allow generalization and inference on the causal relationship between T2DM and alexithymia, but just allows an explorative inspection needing confirmation in further research. In addition, the lack of experimental and evaluation studies does not consent getting exhaustive conclusions about the “true” impact of alexithymia, that could be biased by its interrelations with other relevant aspects of the psychological status. Then, the self-report nature of TAS-20 represents a further limitation because a multi-method approach is recommended in assessing alexithymia since it has been argued that individuals with alexithymic characteristics could not give an accurate estimation of their affective disturbances due to their reduced emotional insight (Lumley et al., 2005).

## Conclusion

In conclusion, this systematic review provides an overview and offer new insights about the clinical need of assessing alexithymia in patients suffering T2DM as its prevalence and strong association with ineffective disease management and psychological distress. Currently, there is empirical evidence which indicates the association between poor glycemic control and psychological distress (e.g., depression, anxiety, quality of life) in patients with T2DM with high alexithymia levels. However, the significance of several aspects remain unclear. Specifically, further studies are needed to improve evidence concerning the role of age, gender, additional diabetes- or treatment-related information, and lifestyle factors, as to provide new research directions.

## Data Availability Statement

The raw data supporting the conclusions of this article will be made available by the authors, without undue reservation.

## Author Contributions

GM made significant contribution to the conception and design of the systematic review, acquisition, qualitative analysis, and synthesis of data by drafting both the first and revised versions of the manuscript. ACap contributed to the acquisition, qualitative analysis, and synthesis of data by drafting the first version of the manuscript. CV and ACat gave significant contribution to draft part of the manuscript. PS and MQ revised manuscript for intellectual content and gave the final approval of the manuscript to be submitted. All authors contributed to the article and approved the submitted version.

## Conflict of Interest

The authors declare that the research was conducted in the absence of any commercial or financial relationships that could be construed as a potential conflict of interest.
